# FSRD: fungal stress response database

**DOI:** 10.1093/database/bat037

**Published:** 2013-06-11

**Authors:** Zsolt Karányi, Imre Holb, László Hornok, István Pócsi, Márton Miskei

**Affiliations:** ^1^Department of Medicine, Medical and Health Science Center, University of Debrecen, H-4032 Debrecen Nagyerdei krt. 98, Hungary, ^2^Institute of Horticulture, University of Debrecen, H-4032 Debrecen Böszörményi út 138, Hungary, ^3^Plant Protection Institute, Centre for Agricultural Research, Hungarian Academy of Sciences, H-1022 Budapest Herman Ottó út 15, Hungary, ^4^Mycology Group of the Hungarian Academy of Sciences, Institute of Plant Protection, Szent István University, H-2103 Gödöllő Páter K. u. 1, Hungary and ^5^Department of Microbial Biotechnology and Cell Biology, University of Debrecen, H-4032 Debrecen Egyetem tér 1, Hungary

## Abstract

Adaptation to different types of environmental stress is a common part of life for today’s fungi. A deeper understanding of the organization, regulation and evolution of fungal stress response systems may lead to the development of novel antifungal drugs and technologies or the engineering of industrial strains with elevated stress tolerance. Here we present the Fungal Stress Response Database (http://internal.med.unideb.hu/fsrd) aimed to stimulate further research on stress biology of fungi. The database incorporates 1985 fungal stress response proteins with verified physiological function(s) and their orthologs identified and annotated in 28 species including human and plant pathogens, as well as important industrial fungi. The database will be extended continuously to cover other fully sequenced fungal species. Our database, as a starting point for future stress research, facilitates the analysis of literature data on stress and the identification of ortholog groups of stress response proteins in newly sequenced fungal genomes.

**Database URL:** http://internal.med.unideb.hu/fsrd

## Introduction

In terms of geological times and evolutionary events, plants taking part in the terrestrialization in the Ordovician–Devonian (∼480–430 Mya) had to face a basically harsh environment with water and nutrient limitations, UV radiation, temperature stress, hostile microbes and the deleterious effects of the oxidative atmosphere (1). Adaptation to this stressful environment altered the metabolism of the pioneer terrestrial plants considerably, affected their cellular and organ diversification, but these events also facilitated the establishment of evolutionarily new plant–microbe symbiotic interactions including the formation of arbuscular mycorrhiza (1–5). It is reasonable to assume that adaptation to the versatile stress conditions including oxidative stress also affected the diversification of terrestrial fungi. In addition, reactive oxygen species are known to play important functions in the development of both land plants (1, 6) and fungi (7–10).

An increasing body of evidence indicates that organisms belonging to the Kingdom of Fungi today are fairly successful in adaptation to a great variety of environmental stress. Fungal cells may acquire resistance to a wide array of impending stress *via* adaptation to mild-stress conditions (11, 12), and their acquired capabilities may even be transmitted to successor cell generations, which never experienced stress (‘cellular memory’) (13). Undoubtedly, acquired stress resistance will strengthen the competitiveness of fungi living in a rapidly changing and stressful environment (11–13). Such powerful and multifaceted stress adaptation tools are needed for parasitic fungi to survive the on-going defense attacks by the host organisms, like plants (14–16) or humans (17–21). Stress adaptation is also an important factor for industrial fungi cultured in bioreactors under stressful conditions to produce valuable goods in high yields (22–24).

**Figure 1.**
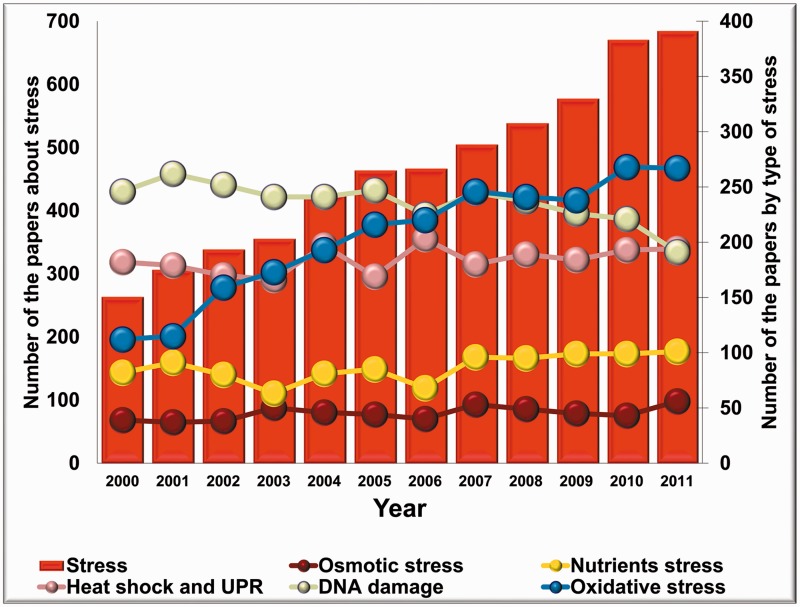
The number of stress-related articles published on fungi from 2000. Columns show the annual number of fungal stress research articles; closed symbols connected by lines represent the number of articles grouped according to selected types of stress (oxidative stress, osmotic stress, nutrients stress, DNA damage, heat shock and unfolded protein response ‘UPR’).

It is worthy to note that the number of stress-related publications on fungi has been increasing uninterruptedly, starting from the early 2000s. Considering the topics, common stress types like osmotic stress, nutrient-deprivation stress, heat shock, DNA damage and oxidative stress have been dominating the majority of the articles published on fungal stress responses in the past decade. Interestingly, the annual number of articles focused on DNA damage and repair decreased over the past few years, whereas the number of reports on osmotic stress, nutrient-deprivation stress and heat shock responses are stagnating. Nevertheless, the number of publications on oxidative stress has been growing remarkably resulting in a significant overall increase in the number of stress-related articles in this eukaryotic kingdom (Figure 1).

Undoubtedly, oxidative stress research has been flourishing both in yeasts and filamentous fungi for several reasons. First of all, successful adaptation to oxidative stress seems to be an indispensible part of invading the host organisms by fungal parasites, and this interesting perception initiated multilevel and diversified research in this field. The baker’s yeast, *Saccharomyces cerevisiae* is regarded as a useful model when human diseases associated with oxidative stress like Alzheimer’s and Parkinson’s diseases (25) or Friedreich’s ataxia (26, 27) are targeted. Important to note that oxidative stress-related metabolic changes may lead to the production of harmful secondary metabolites (28, 29) requesting tight control in various technological processes. Fungal saprophytes also need self-defense against oxidative stress when they produce diffusible oxidizers to disrupt the structure of plant biopolymers, like lignin (30).

In the past decade, the number of wholly sequenced fungal genomes steadily increased. The wealth of information extracted from genome sequencing and annotation data is freely available to the scientific community through databases like the *Saccharomyces* Genome Database (31), the *Candida* Genome Database (32), the PomBase (33), the Central *Aspergillus* Data REpository (C*A*DRE) (34) or the *Aspergillus* Genome Database (AspGD) (35). To facilitate the identification and annotation of stress response genes and proteins, our research team took part in the update of the *Aspergillus nidulans* genome annotation in 2008 (36) and established ASB, the Aspergillus Stress Database (37). Considering the active and on-going interest in stress research in fungi, we decided to make the Fungal Stress Response Database, which incorporates now homology search and annotation data gained for 28 species representing three major phyla in the Kingdom of Fungi (38). We hope that this effort will also stimulate future research aiming at a deeper understanding of organization, regulation and evolution of fungal stress response systems.

## Construction

Fungal stress response proteins were collected from the AmiGO database (http://amigo.geneontology.org/) (39) and *via* literature search in NCBI PubMed (http://www.ncbi.nlm.nih.gov/pubmed). Importantly, no putative stress responsive proteins annotated solely on the basis of sequence homologies were taken into consideration, instead only elements with a genuine functional characterization accessible through PubMed entries were used in this work. Altogether 1848 publications were analyzed in this project. Typical stress responsive proteins included stress signaling and signal transduction proteins, stress responsive transcriptional regulators and gene products of the stress defense systems. As a result of the database and literature search for stress response, as much as 1985 stress-related proteins of the following fungal species was collected: *Aspergillus flavus*, *Aspergillus fumigatus*, *A. nidulans*, *Aspergillus oryzae*, *Candida albicans*, *Candida glabrata*, *Cryptococcus neoformans*, *Fusarium graminearum*, *Fusarium oxysporum*, *Fusarium verticillioides*, *Neosartorya fischeri*, *Neurospora crassa*, *S. cerevisiae*, *Schizosaccharomyces pombe* and *Ustilago maydis* (starter database; Supplementary Table S1).

**Figure 2.**
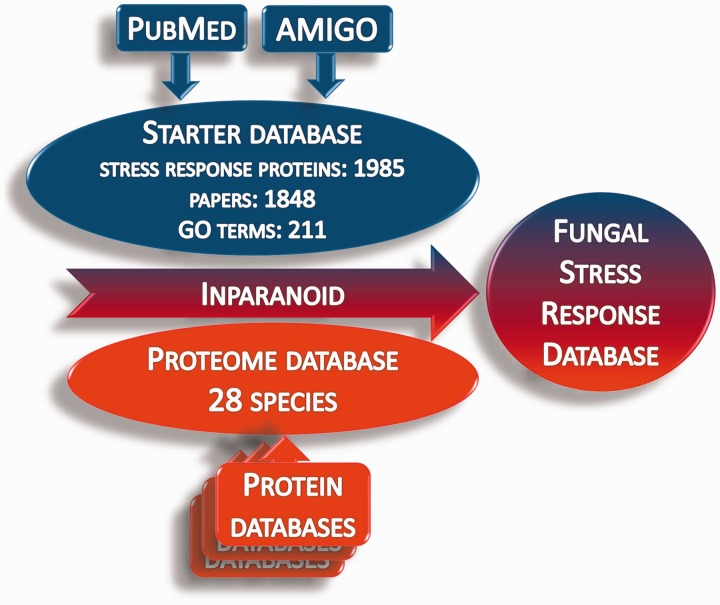
Summary of the construction of the Fungal Stress Response Database. Starter database was constructed from the stress response proteins, which were collected from AmiGO and PubMed databases. Protein sequences in the starter database were used to identify the orthologs in the proteome databases via homology search by the Inparanoid 4.1 software.

**Figure 3.**
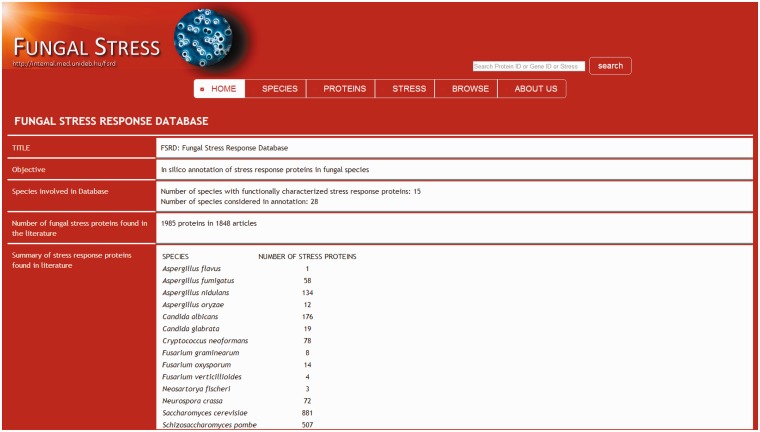
The fungal stress response database entry side.

Whole proteome data for 28 fungus species (proteome database) were obtained from publicly available Internet sources (Supplementary Table S2). The ortholog groups of stress response proteins were identified in the proteome database using the Inparanoid 4.1 software (Figure 2) (40–43). The orthologs were compared by Clustal Omega software (44, 45). The identified orthologs are presented in the Fungal Stress Response Database (Figure 3; http://internal.med.unideb.hu/fsrd), where 29 723 orthologs of the 1985 stress response proteins can be found in 28 species. FSRD is usable with any internet browser. Software consists of the following components:

- On server side: MS SQL server database and IIS webserver;
- On client side: HTML, javascript with jquery.

## Database interface and visualization

In addition to gaining an easy access to basic information on the stress response proteins and their function(s) (GO stress terms, protein sequences, literature data) visitors of the Fungal Stress Response Database can also navigate to the appropriate species-specific databases. The database entry side contains ‘Home’, ‘Species’, ‘Proteins’, ‘Stress types’, ‘Browse’, ‘Search’ and ‘About us’ sections. ‘Home’ section contains general information about this project. ‘Species’ option is the list of fungal species currently covered by the Fungal Stress Response Database. Section ‘Protein’ incorporates all proteins with a verified role in stress response. After clicking on the protein ID, visitors can gain information on the type(s) of stress the actual protein is associated with; there are also links to the species-specific database of the particular protein and to relevant PubMed entries (via PMID numbers) providing further knowledge about the protein’s function. Section ‘Stress types’ summarizes GO stress terms grouped according to the six main stress categories: oxidative stress, osmotic stress, starvation, heat shock and unfolded protein response, DNA damage and other stress. Stress terms are linked to fungal stress response proteins. In ‘Browse’ section, visitors can identify the orthologs of the stress response proteins and visitors can download multiple sequence alignment of orthologs from this site. ‘Search’ option leads to a tool allowing any search based on protein names, IDs, stress terms, etc.

## Conclusion

The selection of other species was based on their biomedical (e.g. *Aspergillus* spp., *Candida* spp., *C. neoformans*), industrial (e.g. *Aspergillus* spp., *S. cerevisiae*, *Yarrowia lipolytica*, *Kluyveromyces lactis*) or agricultural (e.g. fusaria, *Magnaporthe grisea*, *U. maydis*) importance. Species with genuinely identified and characterized stress response proteins like *S. pombe* and *N. crassa* were also preferred. Considering the remarkable progress reached in the past decade in deciphering the stress response systems of fungi, as well as the on-going and foreseeable fungal whole-genome sequencing and annotation efforts, we want to stimulate and facilitate further research in this important field by a continuous revision and expansion of our Fungal Stress Response Database; data on genomes and proteomes of newly sequenced fungal species will be regularly incorporated into the database. Furthermore, we would like to draw the attention of the international mycologist community to filamentous fungus models, like *A. nidulans* and *N. crassa* (36, 46). A multilevel functional analysis of the genome sequences of these fungi would certainly lead to a deeper understanding of the molecular background of stress response systems of all major fungal phyla. Last but not least, such abundance of information on the stress response systems in the Kingdom of Fungi will hopefully help us to understand the stress adaptation processes of other eukaryotes including humans.

## Supplementary Data

Supplementary data are available at *Database* Online.

Supplementary Data
