# Supplementary material for: FSRD: fungal stress response database
Source: Database (Oxford). 2013 Jun 11;2013:bat037. doi: 10.1093/database/bat037 (PMC3678302; doi:10.1093/database/bat037)
Supplement: Supplementary Data [file supp_2013_bat037_index.html]

Supplementary Data 

# FSRD: fungal stress response database

## 

files

**Files in this Data Supplement:**

- Supplementary Data - xls file
- Supplementary Data - xls file
